# Electronic Patient‐Reported Outcome Quality of Life Score in Japanese Patients With Pancreatic Cancer on Second‐Line Chemotherapy: A Multicenter Observational Study

**DOI:** 10.1002/cam4.71161

**Published:** 2025-08-26

**Authors:** Yuki Takumoto, Akihiro Ohba, Takeshi Terashima, Makoto Ueno, Kenji Ikezawa, Naohiro Okano, Takuji Okusaka, Chigusa Morizane, Masafumi Ikeda, Masato Ozaka, Hiroto Narimatsu, Manabu Akazawa, Takeru Shiroiwa, Junji Furuse

**Affiliations:** ^1^ Center for Outcomes Research and Economic Evaluation for Health National Institute of Public Health Saitama Japan; ^2^ Department of Public Health and Epidemiology Meiji Pharmaceutical University Tokyo Japan; ^3^ Department of Hepatobiliary and Pancreatic Oncology National Cancer Center Hospital Tokyo Japan; ^4^ Department of Gastroenterology Kanazawa University Hospital Kanazawa Japan; ^5^ Department of Gastroenterology Kanagawa Cancer Center Kanagawa Japan; ^6^ Department of Hepatobiliary and Pancreatic Oncology Osaka International Cancer Institute Osaka Japan; ^7^ Department of Medical Oncology Kyorin University Faculty of Medicine Tokyo Japan; ^8^ Department of Hepatobiliary and Pancreatic Oncology National Cancer Center Hospital East Kashiwa Japan; ^9^ Gastroenterology Center, Gastroenterological Medicine The Cancer Institute Hospital of JFCR Tokyo Japan; ^10^ Cancer Prevention and Control Division Kanagawa Cancer Center Research Institute Kanagawa Japan

**Keywords:** chemotherapy, electronic patient‐reported outcome, EORTC‐QLQ‐C30, EQ‐5D‐5L, health‐related quality of life, pancreatic cancer, PRO‐CTCAE

## Abstract

**Introduction:**

Patients' QoL scores during chemotherapy are generally measured during hospital visits. However, patients frequently recover from AEs before hospital arrival. This study continuously assessed each chemotherapy's impact on patients' QoL scores during hospital visits and at home using an electronic device (ePRO).

**Methods:**

This multicenter, prospective observational study was conducted in 29 Japanese hospitals. Patients were treated with liposomal irinotecan, fluorouracil, and levoleucovorin (Nal‐IRI + 5‐FU/LV), gemcitabine and nab‐paclitaxel (GnP), or gemcitabine (GEM) as second‐line chemotherapy for unresectable pancreatic cancer. All respondents used ePRO to answer several QoL questionnaires on Days 1, 2, 4, 6, 8, and 11 following chemotherapy administration. The primary endpoint was the EQ‐5D‐5L index value, and the secondary endpoints were AE frequency and the EORTC QLQ‐C30.

**Results:**

The analysis included 67 participants. The mean ± SD QoL scores of the Nal‐IRI + 5‐FU/LV and GnP arms varied from 0.803 ± 0.142 to 0.678 ± 0.247 and 0.872 ± 0.077 to 0.726 ± 0.185, respectively, over the evaluation period. The highest and lowest QoL scores were observed around the chemotherapy administration and approximately 1 week after the chemotherapy administration, respectively, in both arms.

**Conclusions:**

Patients' QoL score assessment over time by ePRO revealed QoL score trends, which emphasized the importance of QoL management outside of hospital visits for each chemotherapy.

## 1 Introduction

Pancreatic cancer (PC) is the leading cause of cancer‐related deaths in developed countries and one of the deadliest cancers globally [1, 2]. The National Comprehensive Cancer Network and clinical practice guidelines for PC in Japan recommended chemotherapy for unresectable PC (UPC), but several treatment options exist [3, 4, 5]. The 2022 clinical practice guidelines for PC in Japan recommend 5‐fluorouracil, leucovorin, irinotecan, and oxaliplatin: FOLFIRINOX (FFX) or the combination of gemcitabine and nab‐paclitaxel (GnP) as a first choice for UPC [5]. Several treatment options exist for secondary chemotherapy based on performance status and previous chemotherapy, including FFX, GnP, the combination of a liposomal formulation of irinotecan, fluorouracil, and levoleucovorin (Nal‐IRI + 5‐FU/LV), gemcitabine (GEM), and S‐1 [6, 7, 8]. Treatment options should avoid overlapping treatment regimens for first‐line chemotherapy. However, treating UPC is difficult even with various treatment options, and maintaining and improving QoL is one of the most important treatment objectives [5].

The impact of regimens on patients' QoL is often investigated using patient‐reported outcome measures (PROMs) [9, 10]. PROMs include the EuroQol 5 Dimensions 5‐Level (EQ‐5D‐5L) index value, the Short Form 6 Dimension (SF‐6D), and the Health Utility Index (HUI) as general indicators [11, 12, 13]. Additionally, the European Organization for Research and Treatment of Cancer QLQ‐C30 (EORTC‐QLQ‐C30) and Functional Assessment of Cancer Therapy‐General (FACT‐G), which can also measure cancer‐specific disease states and adverse events (AEs), are frequently used [14, 15]. Such PROMs are often investigated by patients completing a paper‐based QoL questionnaire during hospital visits [16, 17]. In contrast, AEs in cancer treatment do not necessarily occur on the administration day. In particular, symptoms, such as constipation and diarrhea, occur approximately a week from the day of administration for PC. Moreover, fatigue, neurotoxicity, and myelosuppression persist and worsen or improve in the long term [18, 19, 20, 21, 22]. Therefore, the patient's QoL may not be adequately assessed using paper questionnaires during hospital visits [23, 24].

Studies have recently reported QoL surveys using electronic devices (ePRO) as a solution to paper‐based QoL survey limitations [25, 26]. A previous Japanese study compared two arms of patients with cancer undergoing chemotherapy, one using an ePRO to assess QoL during hospital visits and at home, and the other using a paper‐based survey to assess QoL only during hospital visits [27]. Both arms demonstrated significantly lower QoL values for electronic devices at home, despite similar QoL values in their responses during hospital visits. Assessing QoL scores during hospital visits and at home using ePRO will allow more timely treatment selection for the patient's condition.

These points indicate the importance of assessing the impact of chemotherapy and associated AEs on patients' QoL in treating UPC, where the disease status is severe and chemotherapy‐induced AEs are substantial. However, the detailed effect of chemotherapy on QoL in the second‐line treatment of UPC, where the patient's condition is also severe compared to first‐line treatment, remains unknown; no studies assess the effect of chemotherapy used in the second‐line treatment of UPC on patients' QoL over time, especially at home. Therefore, this longitudinal study assessed patients' QoL during the second chemotherapy period of UPC using an ePRO during hospital visits and at home and closely examined the effect of each chemotherapy on QoL scores during this period.

## 2 Materials and Methods

### 2.1 Study Design

This multicenter prospective observational study assessed the impact of second‐line chemotherapy on QoL scores in PC over time, both during hospital visits and at home. Study participants received or were scheduled to receive one of the prescribed chemotherapies (Nal‐IRI + 5‐FU/LV, GEM, or GnP) as second‐line chemotherapy for UPC in an outpatient setting.

### 2.2 Participants

The chemotherapy regimen prescribed was the usual second‐line treatment regimen for UPC, following the 2022 clinical practice guidelines for PC in Japan. Five oncologists on the study's research review committee guided the selection [5].

Inclusion criteria:

- Patients with a confirmed invasive pancreatic adenocarcinoma diagnosis by cytology and/or histology
- Patients scheduled to receive the second cycle of defined second‐line chemotherapy for unresectable advanced or metastatic PC in an outpatient setting
- Patients who are at least 20 years old at consent
- Patients who can operate ePRO (tablets), including those requiring partial assistance
- Patients who provided written consent to participate in the study

Exclusion criteria:

- Patients deemed unsuitable for study enrollment by the principal investigator or a research assistant.

### 2.3 Procedures

All study participants used tablets to complete EQ‐5D‐5L, EORTC‐QLQ‐30, and PRO‐Common Terminology Criteria for Adverse Events (CTCAE) questionnaires [11, 14, 28].

The EQ‐5D‐5L is a QoL questionnaire with five attributes (“mobility,” “self‐care,” “usual activities,” “pain/discomfort,” and “anxiety/depression”), each rated by the patient on a 5‐point scale. An algorithm based on general population social thinking in Japan converted responses into EQ‐5D‐5L index values [29, 30]. The EQ‐5D‐5L index values are calculated on a scale from 1, indicating a state of perfect health, to 0, indicating a state of death. The EORTC‐QLQ‐C30 is a disease‐specific measure for cancer consisting of 30 questions [14]. The responses to the EORTC‐QLQ‐C30 questions are tabulated as five function scales (physical, role, emotional, cognitive, and social), nine symptom scales (fatigue, nausea/vomiting, pain, dyspnea, insomnia, anorexia, constipation, diarrhea, and economy), and global health status. Each score is summed on a scale from 0 to 100, with higher values for function scales and global health score and lower values for the symptom scale, indicating a good QoL. The PRO‐CTCAE uses the CTCAE, which is frequently used to assess AEs in cancer clinical trials and is a subjective assessment by patients, to provide a more accurate and precise assessment of adverse event severity [28, 31, 32]. Each study can partially use the PRO‐CTCAE to investigate the severity and impact on life in the past week for each AE. This study assumed 10 AEs (stomatitis, anorexia, nausea, vomiting, constipation, diarrhea, abdominal pain, hand‐foot syndrome, pain, and fatigue) to occur more frequently during chemotherapy for UPC (following the 2022 JPC guidelines) and investigated the greater impact on patient QoL.

The survey date was set as the starting point (Cycle 2 Administration 1 Day 1: C2A1D1) at the first dose of the second cycle in the second‐line chemotherapy. This is because treatment after the second cycle is generally provided on an outpatient basis, where safety management and changes in patient QoL are of particular concern. Survey dates for EQ‐5D‐5L and EORTC‐QLQ‐30 were Days 1, 2, 4, 6, 8, 11, and 15, and every week after that until the next chemotherapy administration date. The survey date for PRO‐CTCAE was every week from C2A1D1. All survey forms on C2A1D1 were completed before chemotherapy, and the survey lasted for two consecutive cycles starting on C2A1D1. Patient background information was collected at the baseline time point, screen time (C1A1D0). Table S1 shows the specific response schedule for each of these arms.

Tablets were lent to study participants after providing informed consent. Instructions on use and practice answers were provided before the first answer. Reminder notifications were provided on the tablet on each answer date, and a system was established to enable research collaborators to contact the support center.

### 2.4 Statistical Analysis

The number of cases in which EQ‐5D‐5L‐derived results are expected to converge to a specific value is approximately 50 per arm based on a previous study [27]. Therefore, the target number of enrolled cases was a maximum 150 for the three arms. Meanwhile, the target patient population for this study is limited in Japan. In addition, the study objectives do not necessarily require a clear sample size design. Due to these two reasons, we considered that we would carry out the analysis even with a small sample size. The analysis selected patients who responded to C2A1D1 with at least one of each measure among the eligible patients. The GEM arm was treated as a reference because of the small sample size, which would have increased uncertainty in the endpoints.

In this study, we calculated descriptive statistics for the important endpoints to evaluate the impact of the second‐line treatment regimen on the QoL of UPS patients. The primary endpoint was the EQ‐5D‐5L index value. Secondary endpoints were each score and the type and severity of PRO‐CTCAE‐derived AE categories. Descriptive statistics were calculated using the scores by time point of assessment. The minimal clinically important difference (MCID) for the EQ‐5D‐5L index and EORTC‐QLQ‐C30 scores was set at 0.05 and 0.1 points, and 10 points based on previous oncology and lung cancer studies, respectively [33, 34]. A variable effect modeling estimation was performed with a predefined adjustment factor to calculate the adjusted mean QoL score for each treatment regimen over the entire study period. SAS version 9.4 (SAS Institute, Cary, NC, USA) and R version 4.2.3 were used for all analyses.

## 3 Results

### 3.1 Participant Background and Answer Rate

**FIGURE 1:**
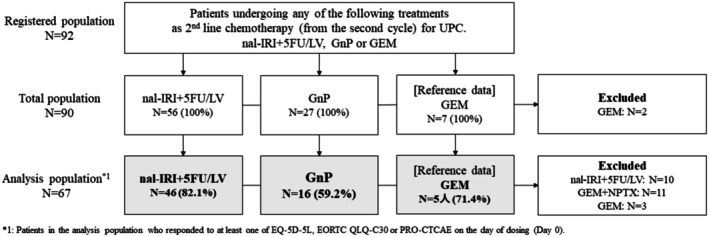
Patient Flow diagram. EORTC‐QLQ‐C30: The European Organization for Research and Treatment of Cancer QLQ‐C30; EQ‐5D‐5L: EuroQol 5 Dimensions 5‐Level; GEM: Gemcitabine; GnP: Gemcitabine + nab‐paclitaxel; Nal‐IRI + 5‐FU/LV: Liposomal irinotecan + 5‐fluorouracil and leucovorin; PRO‐CTCAE: Patient‐Reported Outcome‐Common Terminology Criteria for Adverse Events. UPC: Unresectable pancreatic cancer.

Of the 67 participants analyzed, 46 received Nal‐IRI + 5FU/LV, and 16 received GnP (Figure 1). Table 1 shows their patient background information. Differences between arms indicated a possible bias in gender, age, Eastern Cooperative Oncology Group Performance Status (ECOG PS), and history of first‐line chemotherapy treatment. Table S1 shows the background of the five patients in the GEM arm.

**TABLE 1:** Patient characteristics in the Nal‐IRI + 5‐FU/LV arm and GnP arm.

Characteristics	Nal‐IRI + 5‐FU/LV		GnP		*p*
	*N*	%	*N*	%	
Initial response population	46	—	16	—	—
Sex					
Male	22	47.8%	9	56.3%	0.562
Female	24	52.2%	7	43.8%	
Age, Mean (SD), year	69.1	(8.9)	64.8	(7.7)	0.088
BMI, Mean (SD), kg/m^2^	21.5	(3.2)	21.5	(3.2)	0.334
ECOG PS					
0	20	43.5%	12	75.0%	< 0.01
1	26	56.5%	4	25.0%	
2~	0	0.0%	0	0.0%	
History of surgery					
No	37	80.4%	13	81.3%	0.629
Yes	9	19.6%	3	18.8%	
History of radiotherapy					
No	44	95.7%	15	93.8%	0.839
Yes	2	4.3%	1	6.3%	
History of preoperative chemotherapy					
No	42	91.3%	15	93.8%	0.615
Yes	4	8.7%	1	6.3%	
History of postoperative chemotherapy					
No	40	87.0%	14	87.5%	0.663
Yes	6	13.0%	2	12.5%	
First‐line chemotherapy					
(modified) FOLFIRINOX	1	2.2%	12	75.0%	< 0.01
GnP	40	87.0%	0	0.0%	
GEM	4	8.7%	0	0.0%	
S‐1	0	0.0%	0	0.0%	
Other	1	2.2%	4	25.0%	
First‐line chemotherapy, mean (SD), Day	266.8	(186.8)	203.9	(120.5)	0.214

Abbreviations: ECOG: Eastern cooperative oncology group; GEM: gemcitabine; GnP: gemcitabine + nab‐paclitaxel; Nal‐IRI + 5‐FU/LV: liposomal irinotecan + 5‐fluorouracil and leucovorin; PS: Performance status.

The answer rate for the Nal‐IRI + 5‐FU/LV arm in the analysis population remained ≥ 80.4% within 14 days of the first dose of each cycle (Table S2). The answer rate for the GnP arm remained > 68.8% in both arms within 28 days of the date of the first dose of each cycle.

### 3.2 EQ‐5D‐5L Index Values

The adjusted mean QoL scores (95% confidence interval) for patient background factors based on the EQ‐5D‐5L index score were 0.714 (0.688 ~ 0.739), 0.709 (0.684 ~ 0.734), and 0.799 (0.769 ~ 0.829) in the NAL‐IRI + 5FU/LV, GnP, and GEM arms, respectively.

**FIGURE 2:**
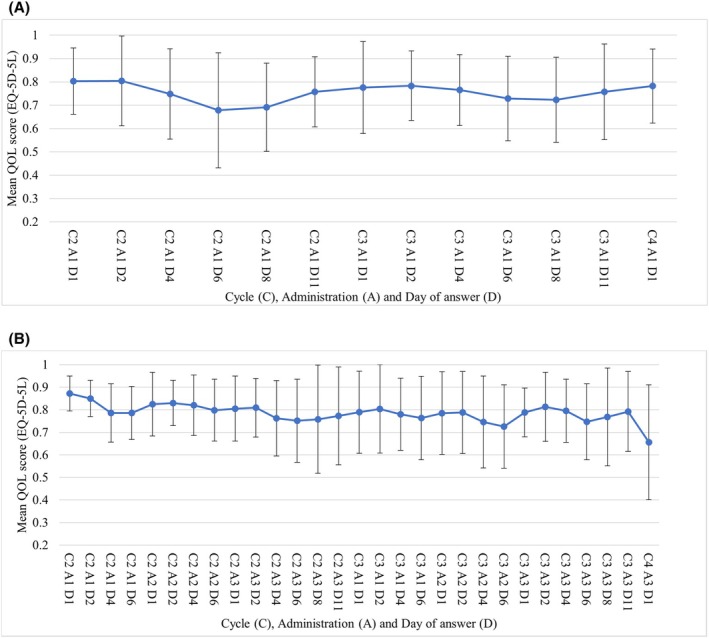
QoL scores from EQ‐5D‐5L index values. (A) Nal‐IRI + 5‐FU/LV. (B) GnP EQ‐5D‐5L: EuroQol 5 Dimensions 5‐Level; QoL: Quality of life.

The mean QoL score ± standard deviation (SD) in the NAL‐IRI + 5FU/LV arm decreased from C2A1D1 to C2A1D6 from 0.803 ± 0.142 to 0.678 ± 0.247 and recovered by 0.098 points to 0.776 ± 0.182 in C3A1D1 (Figure 2, Table S3). This trend remained similar until C4A1D1. The mean QoL score ± SD in the GnP arm at C2A3D6 was 0.751 ± 0.184, which is the lowest value in Cycle 2. However, the QoL score at C3A1D1 was 0.789 ± 0.182, which is a smaller improvement in the QoL score from the lowest value compared to the NAL‐IRI + 5FU/LV arm (recovery range: 0.048 points). This trend was similar for the percentage of patients whose QoL scores decreased beyond the MCID (Figure S1).

### 3.3 EORTC‐QLQ C30

**FIGURE 3:**
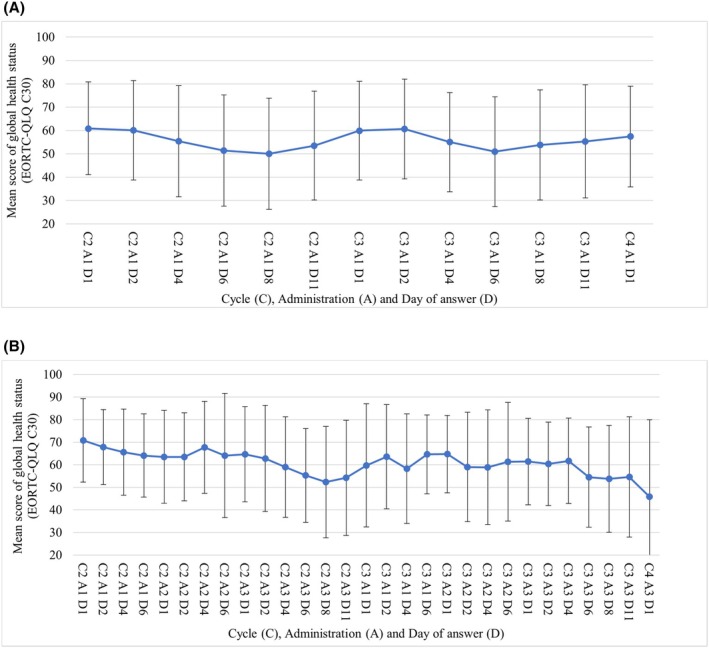
EORTC‐QLQ C30 global health status. (A) Nal‐IRI + 5‐FU/LV. (B) GnP. EORTC‐QLQ‐C30: The European Organization for Research and Treatment of Cancer QLQ‐C30.

Global health status trends of EORTC‐QLQ C30 were similar to that of the EQ‐5D‐5L‐derived QoL scores, with the mean score increasing or decreasing following the date of chemotherapy administration (Figure 3, Table S4). The mean score ± SD in the NAL‐IRI + 5FU/LV arm decreased from 60.9 ± 19.9 in C2A1D1 to 50.0 ± 23.8 in C2A1D8 and recovered 10.6 points to 60.6 ± 21.4 in C3A1D1. This trend remained similar until C4A1D1, with the GnP arm having the lowest mean score ± SD in C2A3D8 at 52.3 ± 24.7. However, the subsequent score on C3A1D1 was 59.7 ± 27.3, with a smaller improvement in score from the lowest value compared to the NAL‐IRI + 5FU/LV arm (recovery range: 7.4 points).

**FIGURE 4:**
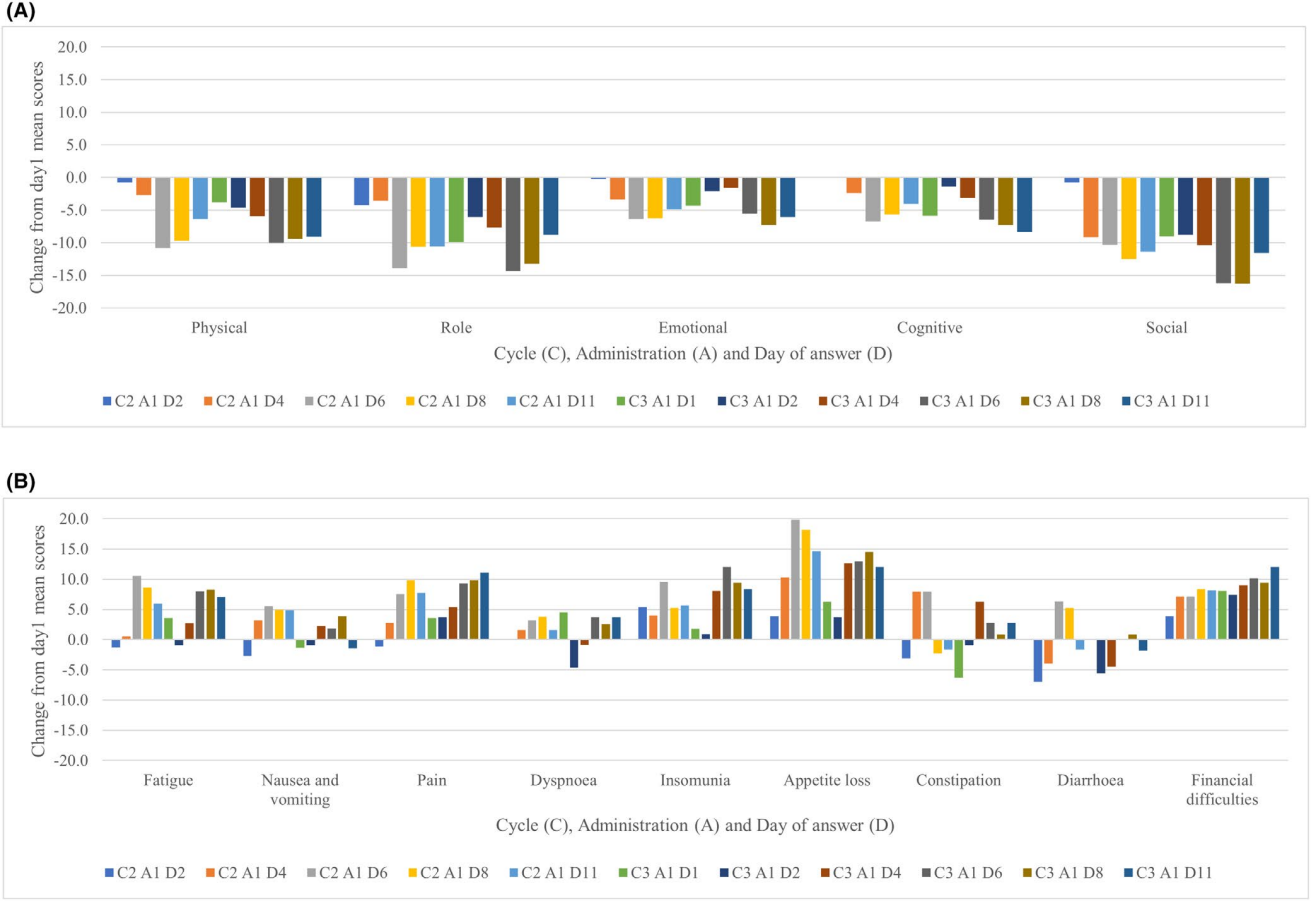
Change from Day 1 of function scales and symptom scales. (A) Functional scales of EORTC‐QLQ‐C30 in Nal‐IRI + 5‐FU/LV. (B) Symptom scales of EORTC‐QLQ‐C30 in Nal‐IRI + 5‐FU/LV.

Figure 4 shows the changes in function and symptom scales. The factors whose mean scores decreased beyond the set MCID were physical, role, and social functions in the functional scales, and fatigue, pain, insomnia, appetite loss, and financial difficulties in the symptom scales in the NAL‐IRI + 5FU/LV arm. Financial difficulties continued to have an increasing impact after the washout period in the NAL‐IRI + 5FU/LV arm. However, the factors with a mean score decline exceeding the established MCID were physical, role, and cognitive functions in the functional scales, and pain, insomnia, constipation, diarrhea, and cognitive function in the symptom scales in the GnP arm. Of these factors, physical, role, and cognitive functions and pain demonstrated an increasing trend in impact after a washout period in the GnP arm.

### 3.4 PRO‐CTCAE

Over 30% of reported symptoms (pain, anorexia, diarrhea, abdominal pain, and fatigue) in the NAL‐IRI + 5FU/LV arm were classified as Grade 3–4 (Table 2). A similar trend was observed in the GnP arm, but the proportion of Grade 3–4 AEs was lower.

**TABLE 2:** Summary of adverse events based on PRO‐CTCAE.

Adverse event	Nal‐IRI + 5‐FU/LV (*N* = 46)				GnP (*N* = 17)				GEM^a^ (*N* = 5)			
	All grade		Grades 3–4		All grade		Grades 3–4		All grade		Grades 3–4	
	*N*	%	*N*	%	*N*	%	*N*	%	*N*	%	*N*	%
Oral pain	28	60.9%	2	4.3%	12	76.5%	0	0%	1	20.0%	0	0%
Anorexia	43	93.5%	14	30.4%	15	94.1%	4	23.5%	5	100%	1	20.0%
Nausea	37	80.4%	6	13.0%	8	52.9%	0	0%	3	60.0%	0	0%
Vomiting	17	37.0%	3	6.5%	4	23.5%	0	0%	2	40.0%	0	0%
Constipation	38	82.6%	5	10.9%	16	100%	3	17.6%	5	100%	0	0%
Diarrhea	36	78.3%	17	37.0%	13	82.4%	4	23.5%	3	60.0%	0	0%
Abdominal pain	39	84.8%	15	32.6%	14	88.2%	3	17.6%	5	100%	1	20.0%
Hand–foot syndrome	26	56.5%	4	8.7%	9	58.8%	3	17.6%	1	20.0%	0	0%
Pain	43	93.5%	20	43.5%	16	100%	4	23.5%	5	100%	0	0%
Fatigue	44	95.7%	14	30.4%	16	100%	4	23.5%	5	100%	1	20.0%

Abbreviations: GEM: gemcitabine; GnP: gemcitabine + nab‐paclitaxel; Nal‐IRI + 5‐FU/LV: liposomal irinotecan + 5‐fluorouracil and leucovorin.

^a^ The GEM arm was the reference's result.

## 4 Discussion

We conducted a multicenter prospective observational study of Japanese UPC patients undergoing the Nal‐IRI + 5‐FU/LV, GnP, or GEM as second‐line chemotherapy, using ePRO to investigate QoL longitudinally. With regard to patient characteristics, the peak age of patients with PC in Japan is approximately 75 years [35]. Patient characteristics in this study are generally similar, although the average age is slightly younger. The mean age was younger in the GnP arm than in the Nal‐IRI + 5‐FU/LV arm, and the proportion of patients with ECOG PS of 0 was higher. These indicate that the GnP arm may have been patients with relatively unimpaired health status that could better tolerate the intense treatment.

The mean adjusted QoL score derived from EQ‐5D‐5L was similar to the QoL score (after pharmaceutical intervention: 0.74) of patients with advanced PC in a previous study [36], consistent with this study. The QoL score evolution derived from the EQ‐5D‐5L showed a decline in the Nal‐IRI + 5‐FU/LV arm until Day 6 (from C2A1D1 to C2A1D6) postdose. Then, a recovery trend was observed in the week leading up to the following dosing day (from C2A1D6 to C3A1D1) (Figure 2). This change indicates lower QoL values at home in the Nal‐IRI + 5‐FU/LV arm than at the hospital visit and tended to recover to the same level as QoL values at C2A1D1 after a washout period. In contrast, the QoL score in the GnP arm was lower at home than at the hospital visit as in the Nal‐IRI + 5‐FU/LV arm, but the mean QoL score indicated a trend toward insufficient recovery during the washout period. This could be because the GnP arm may not have recovered enough from the disutility of chemotherapy, compared to the Nal‐IRI + 5‐FU/LV arm, which is administered once every 2 weeks, while the GnP arm is repeated once every 3 weeks. Some patients' QoL scores decreased beyond the MCID relative to the initial response date (C2A1D1). The treatment regimen was indicated to have no small impact on the patient's QoL at home (Figure S1).

The trends for each treatment regimen were similar to those in Figure 2 above; although the global health values were lower in absolute score than the QoL scores derived from the EQ‐5D‐5L (Figure 3). The similarity of the result trends of both measures ensures the consistency of the assessment methods of both instruments, although both measures differ in the format of the questions.

The function scale in the Nal‐IRI + 5‐FU/LV arm demonstrated recovery after a washout period, although some elements may temporarily exceed the value of the established MCID. However, role and social functions repeatedly worsened beyond the MCID with treatment. Therefore, the physical and mental symptoms associated with Nal‐IRI + 5‐FU/LV may impact daily family and work activities, indicating the need for these factors to be closely monitored. Symptom scales in the Nal‐IRI + 5‐FU/LV arm demonstrated that the impact of several AEs, including appetite loss, repeatedly occurred immediately after administration and during the washout period beyond the MCID. Financial difficulties continued to worsen, but without exceeding the MCID. Eighty‐seven percent of patients in the Nal‐IRI + 5‐FU/LV arm undergo more expensive GnP than Nal‐IRI + 5‐FU/LV as first‐line chemotherapy, causing a cumulative financial burden. Therefore, patients were suggested to be aware of the potential financial concerns with administering Nal‐IRI + 5‐FU/LV as second‐line chemotherapy for UPC.

Function scales in the GnP arm revealed that physical, role, and cognitive functions worsened over time beyond the MCID. Symptoms in the symptom scales in the Nal‐IRI + 5‐FU/LV arm worsened to a certain extent after the washout period; however, pain symptoms were more intense in the third cycle. Constipation, which was rarely reported in the Nal‐IRI + 5‐FU/LV arm, was reported in the GnP arm. These findings indicate the importance of controlling pain and constipation at home when administering GnP and the need to pay attention to the effects on physical and cognitive functions in daily life.

Some symptoms associated with the function and symptom scales in PRO‐CTCAE were confirmed (Table 2). For example, the Nal‐IRI + 5‐FU/LV arm had higher rates of grade 3–4 pain (oral, abdominal, and aching) and fatigue than the GnP arm. These may have had an impact above the MCID on symptom scales such as fatigue, pain, and insomnia. The GnP arm had higher rates of grade 3–4 constipation and hand‐foot syndrome than the IRI + 5‐FU/LV arm, and the constipation symptom scale tended to exceed the MCID.

## 5 Limitations

This study has several study limitations. First, the patient's background demonstrated a bias, making it difficult to assign a preference for the impact of the treatment regimen on QoL and its extent, because this is not a randomized clinical study. However, change trends over time for point estimates may be identified by adjusting for patient characteristics in each arm. Hence, we could identify the impact of individual treatment regimens on QoL values using ePRO, which incorporates multiple questionnaires in this study.

Second, assessing long‐term QoL scores and their changes for all chemotherapy as second‐line chemotherapy was difficult. Although a variety of chemotherapies are available as second‐line chemotherapy in Japan, most used in clinical practice are included in this study. Additionally, there was concern in patients' conditions after the second cycle, as more patients were outpatients. Therefore, this study assessed the impact on QoL scores in the second and third cycles of second‐line chemotherapy (Nal‐IRI + 5‐FU/LV, GnP or GEM) for treating UPC. The average response time was approximately 1.5 months, even in the GnP arm, with the longest standard turnaround time per cycle. This makes the estimation of the long‐term impact on QoL scores difficult. However, the results for each treatment regimen revealed the increasing and decreasing QoL values of the patients (Figures 2 and 3). Thus, we could predict trends in QoL values concerning chemotherapy and the factors that might influence them, although we could not collect long‐term data.

Thirdly, it is possible that the PRO‐CTCAE responses do not clearly indicate the AEs of the day on which patients responded. The PRO‐CTCAE questionnaire investigates symptoms from the day of response to 7 days prior, so it does not indicate all the AEs of the patients on the day of answer. However, as it evaluates AEs up to 7 days prior, even if the response was on the first day of the second cycle, it is thought that it does not deny the impact of the intervention effect of each arm on QoL, because it is during the first cycle of each arm.

The study allowed us to determine the impact of second‐line chemotherapy on QoL scores over time in UPC in Japan, as well as the characteristics of each treatment regimen and the individual impact it may have on QoL scores. This study indicates that regardless of the treatment regimen, second‐line chemotherapy for UPC may reduce QoL levels at home. However, its disutility cannot be said to be fully recovered. The impact of the treatment regimen on the patient's QoL, even at home, is important to be considered. The results of this study will help to understand patients' condition and select and administer appropriate second‐line chemotherapy for UPC.

## 6 Conclusion

QoL score assessment over time by ePRO revealed patterns of QoL score trends, emphasizing the importance of managing QoL outside of hospital visits for each regimen. These trends provide useful information for chemotherapy management.

## Author Contributions

**Yuki Takumoto:** conceptualization (lead), formal analysis (equal), methodology (equal), project administration (lead), software (lead), writing – original draft (lead), writing – review and editing (equal). **Akihiro Ohba:** investigation (equal), methodology (equal), resources (equal), writing – review and editing (equal). **Takeshi Terashima:** investigation (equal), methodology (equal), resources (equal), writing – review and editing (equal). **Makoto Ueno:** conceptualization (equal), investigation (equal), methodology (equal), writing – review and editing (equal). **Kenji Ikezawa:** conceptualization (equal), investigation (equal), methodology (equal), writing – review and editing (equal). **Naohiro Okano:** investigation (equal), methodology (equal), resources (equal), writing – review and editing (equal). **Takuji Okusaka:** conceptualization (equal), investigation (equal), methodology (equal), writing – review and editing (equal). **Chigusa Morizane:** investigation (equal), methodology (equal), writing – review and editing (equal). **Masafumi Ikeda:** conceptualization (equal), investigation (equal), methodology (equal), writing – review and editing (equal). **Masato Ozaka:** conceptualization (equal), investigation (equal), methodology (equal), resources (equal), writing – review and editing (equal). **Hiroto Narimatsu:** conceptualization (equal), methodology (equal), writing – review and editing (equal). **Manabu Akazawa:** conceptualization (equal), methodology (equal), project administration (equal), supervision (equal), writing – review and editing (equal). **Takeru Shiroiwa:** conceptualization (equal), funding acquisition (lead), methodology (equal), supervision (equal), writing – review and editing (equal). **Junji Furuse:** conceptualization (equal), investigation (equal), methodology (equal), project administration (equal), supervision (equal), writing – review and editing (equal).

## Disclosure

Permission to Reproduce Material From Other Sources: Consideration is given to whether permission should be granted in consultation with the authors.

## Ethics Statement

This study was approved by an independent ethics committee for each participating site. All the participants provided written informed consent. We obtained informed consent from all responders before they participated in this study. The independent ethics committees were “Seirei Hamamatsu General Hospital, Clinical Research Review Committee,” “Ethics Review Board of Osaka International Cancer Institute,” “Kyushu University Hospital Institutional Review Board for Clinical Trials,” “Kanazawa University Medical Ethics Review Committee,” “Kanagawa Cancer Center Research Ethics Review Committee,” “National Cancer Center Ethics Committee,” “Faculty of Medicine Research Ethics Committee, Kyorin University,” “Ethics Committee for Observational Studies of Chiba University Hospital,” “Aichi Cancer Center Institutional Review Board,” “Ethics Committee, Tohoku University Graduate School of Medicine,” “Research Ethics Review Committee of the National Cancer Center Hospital East,” “Ethics Committee of Yamagata Prefectural Central Hospital,” “Ethical Review Committee of International University of Health and Welfare Atami Hospital,” “Research Ethics Committee of Kochi Health Sciences Center,” “Ethics Committee, Kindai University Faculty of Medicine,” “Ethics committee, Kagawa University Faculty of Medicine,” “Ethics Committee of Kagoshima University Hospital,” “Sapporo Medical University Ethics Committee,” “Ethical Review Boards in Hyogo Cancer Center,” “Fukushima Medical University Research Ethics Committee,” “Mie University Hospital Clinical Research Ethics Review Committee,” “Ethics Review Board, Chiba Cancer Center,” “Ethics Review Board of Niigata Cancer Center Hospital,” and “National Hospital Organization Kyushu Cancer Center Ethics Committee.”

## Consent

All participants signed an informed consent statement prior to participation in the study.

## Conflicts of Interest

The authors declare no conflicts of interest.

## Supporting information


**Data S1:** cam471161‐sup‐0001‐DataS1.docx.

## Data Availability

Our data are available only to the Center for Outcomes Research and Economic Evaluation for Health, National Institute of Public Health, Saitama, Japan.
