# Supplementary material for: Electronic Patient‐Reported Outcome Quality of Life Score in Japanese Patients With Pancreatic Cancer on Second‐Line Chemotherapy: A Multicenter Observational Study
Source: Cancer Med. 2025 Aug 26;14(17):e71161. doi: 10.1002/cam4.71161 (PMC12378663; doi:10.1002/cam4.71161)
Supplement: Supplementary file 1 — Data S1: cam471161‐sup‐0001‐DataS1.docx. [file CAM4-14-e71161-s001.docx]

**Supplementary Material**

Authors:

Yuki Takumoto, Akihiro Ohba, Takeshi Terashima, Makoto Ueno, Kenji Ikezawa, Naohiro Okano, Takuji Okusaka, Chigusa Morizane, Masafumi Ikeda, Masato Ozaka, Hiroto Narimatsu, Manabu Akazawa, Takeru Shiroiwa, Junji Furuse

Title: Electronic patient-reported outcome quality of life score in Japanese patients with pancreatic cancer on second-line chemotherapy: A multicenter observational study

eTable 1: Research schedule

eTable 2: Patient characteristics in GEM arm

eTable 3: Answer rate for each questionnaire

eTable 4: Detail of QoL scores from EQ-5D-5L index values

eTable 5: Detail of EORTC-QLQ C30 global health status

eFigure 1: Proportion of participant with a reduction in QoL scores from EQ-5D-5L index values on day0 above MICD

eTable 1. Research schedule

1. Nal-IRI+5-FU/LV

| **Cycle (C)** | 1 | 2/3 | | | | | | | | | | | | | | 4 | |
| --- | --- | --- | --- | --- | --- | --- | --- | --- | --- | --- | --- | --- | --- | --- | --- | --- | --- |
| **Cycle day** | 0 | 1 | 2 | 3 | 4 | 5 | 6 | 7 | 8 | 9 | 10 | 11 | 12 | 13 | 14 | 1 | 2~ |
| **Administration (A)** |  | 1 |  |  |  |  |  |  |  |  |  |  |  |  |  | 1 |  |
| **Day of answer (D)^*1^** | 0 | 1 | 2 |  | 4 |  | 6 |  | 8 |  |  | 11 |  |  |  | 15 |  |
| **Characteristics** | X |  |  |  |  |  |  |  |  |  |  |  |  |  |  |  |  |
| **EQ-5D-5L** | X | X^*2^ | X |  | X |  | X |  | X |  |  | X |  |  |  | X^*3^ |  |
| **EORTC-QLQ-C30** | X | X^*2^ | X |  | X |  | X |  | X |  |  | X |  |  |  | X^*3^ |  |
| **PRO-CTCAE** | X | X^*2^ |  |  |  |  |  |  | X |  |  |  |  |  |  | X^*3^ |  |

1. GnP or GEM

| **Cycle (C)** | 1 | 2 | | | | | | | | | | | | | | |
| --- | --- | --- | --- | --- | --- | --- | --- | --- | --- | --- | --- | --- | --- | --- | --- | --- |
| **Cycle day** | 0 | 1 | 2 | 3 | 4 | 5 | 6 | 7 | 8 | 9 | 10 | 11 | 12 | 13 | 14 |  |
| **Administration (A)** |  | 1 |  |  |  |  |  |  | 2 |  |  |  |  |  |  |  |
| **Day of answer (D)^*1^** | 0 | 1 | 2 | 3 | 4 | 5 | 6 | 7 | 1 | 2 | 3 | 4 | 5 | 6 | 7 |  |
| **Characteristics** | X |  |  |  |  |  |  |  |  |  |  |  |  |  |  |  |
| **EQ-5D-5L** | X | X^*2^ | X |  | X |  | X |  | X^*2^ | X |  | X |  | X |  |  |
| **EORTC-QLQ-C30** | X | X^*2^ | X |  | X |  | X |  | X^*2^ | X |  | X |  | X |  |  |
| **PRO-CTCAE** | X | X^*2^ |  |  |  |  |  |  | X^*2^ |  |  |  |  |  |  |  |

B) GnP or GEM (Continued)

| **Cycle (C)** | 3 | | | | | | | | | | | | | | 4 | |
| --- | --- | --- | --- | --- | --- | --- | --- | --- | --- | --- | --- | --- | --- | --- | --- | --- |
| **Cycle day** | 15 | 16 | 17 | 18 | 19 | 20 | 21 | 22 | 23 | 24 | 25 | 26 | 27 | 28 | 1 | 2~ |
| **Administration(A)** | 3 |  |  |  |  |  |  |  |  |  |  |  |  |  | 1 |  |
| **Day of answer (D)^*1^** | 1 | 2 | 3 | 4 | 5 | 6 | 7 | 8 | 9 | 10 | 11 | 12 | 13 | 14 | 15 |  |
| **Characteristics** |  |  |  |  |  |  |  |  |  |  |  |  |  |  |  |  |
| **EQ-5D-5L** | X^*2^ | X |  | X |  | X |  | X |  |  | X |  |  |  | X^*3^ |  |
| **EORTC-QLQ-C30** | X^*2^ | X |  | X |  | X |  | X |  |  | X |  |  |  | X^*3^ |  |
| **PRO-CTCAE** | X^*2^ |  |  |  |  |  |  | X |  |  |  |  |  |  | X^*3^ |  |

EQ-5D-5L, EuroQol 5 Dimensions 5-Level; EORTC QLQ-C30, The European Organization for Research and Treatment of Cancer QLQ-C30; PRO-CTCAE, Patient-Reported Outcome-Common Terminology Criteria for Adverse Events

*1 Except for answers on the day of chemotherapy administration, if the participant fails to answer on the defined date, he/she will be allowed to answer until the day before the next defined date.

*2 Questionnaires are to be completed no later than before the start of chemotherapy administration.

*3 After the last administration date of the third cycle, participants will continue to complete the questionnaire until the start of the fourth cycle in the standard cycle set in the treatment regimen.

eTable 2: Patient characteristics in GEM arm

| Characteristics | GEM | |
| --- | --- | --- |
|  | **N** | **%** |
| Analysis population | 5 | - |
| Sex |  |  |
| Male | 5 | 100% |
| Female | 0 | 0% |
| Age, Mean (SD), year | 72 | (13.7) |
| BMI, Mean (SD), kg/m^2 | 22.3 | (2.9) |
| ECOG PS |  |  |
| 0 | 1 | 20% |
| 1 | 2 | 40% |
| 2~ | 2 | 40% |
| History of surgery |  |  |
| No | 5 | 100% |
| Yes | 0 | 0% |
| History of radiotherapy |  |  |
| No | 5 | 100% |
| Yes | 0 | 0% |
| History of preoperative chemotherapy |  |  |
| No | 5 | 100% |
| Yes | 0 | 0% |
| History of post-operative chemotherapy |  |  |
| No | 5 | 100% |
| Yes | 0 | 0% |
| 1st line chemotherapy |  |  |
| (modified) FOLFIRINOX | 3 | 60% |
| GnP | 0 | 0% |
| GEM | 0 | 0% |
| S-1 | 1 | 20% |
| Other | 1 | 20% |
| 1st line chemotherapy, Mean (SD), Day | 101.2 | (116.1) |

GEM, Gemcitabine; ECOG, Eastern Cooperative Oncology Group; PS, Performance Status; GnP, Gemcitabine + nab-paclitaxel

eTable 3: Answer rate for each questionnaire

A) EQ-5D-5L or EORTC-QLQ-C30

| Cycle | Cycle Day | nal-IRI+5-FU/LV | | GnP | | GEM | |
| --- | --- | --- | --- | --- | --- | --- | --- |
|  |  | N | % | N | % | N | % |
| 2 | 1 | 46 | 100.0% | 16 | 100.0% | 5 | 100.0% |
|  | 2 | 44 | 95.7% | 15 | 93.8% | 5 | 100.0% |
|  | 4 | 42 | 91.3% | 15 | 93.8% | 4 | 80.0% |
|  | 6 | 42 | 91.3% | 16 | 100.0% | 5 | 100.0% |
|  | 8 | 44 | 95.7% | 13 | 81.3% | 4 | 80.0% |
|  | 11 | 42 | 91.3% | 16 | 100.0% | 5 | 100.0% |
|  | 15 | 15 | 32.6% | 12 | 75.0% | 4 | 80.0% |
|  | 22 | 3 | 6.5% | 11 | 68.8% | 1 | 20.0% |
|  | 29 | 1 | 2.2% | 2 | 12.5% | - | - |
| 3 | 1 | 37 | 80.4% | 12 | 75.0% | 2 | 40.0% |
|  | 2 | 37 | 80.4% | 11 | 68.8% | 1 | 20.0% |
|  | 4 | 37 | 80.4% | 13 | 81.3% | 3 | 60.0% |
|  | 6 | 37 | 80.4% | 12 | 75.0% | 1 | 20.0% |
|  | 8 | 40 | 87.0% | 13 | 81.3% | 2 | 40.0% |
|  | 11 | 37 | 80.4% | 14 | 87.5% | 2 | 40.0% |
|  | 15 | 28 | 60.9% | 11 | 68.8% | 2 | 40.0% |
|  | 22 | 7 | 15.2% | 11 | 68.8% | 3 | 60.0% |
|  | 29 | 2 | 4.3% | 6 | 37.5% | 2 | 40.0% |

nal-IRI+5-FU/LV, liposomal irinotecan + 5-fluorouracil and leucovorin; GnP, Gemcitabine + nab-paclitaxel; GEM, Gemcitabine

B) PRO-CTCAE

| Cycle | Cycle Day | nal-IRI+5-FU/LV | | GnP | | GEM | |
| --- | --- | --- | --- | --- | --- | --- | --- |
|  |  | N | % | N | % | N | % |
| 2 | 1 | 46 | 100.0% | 16 | 100.0% | 5 | 100.0% |
|  | 8 | 44 | 95.7% | 13 | 81.3% | 4 | 80.0% |
|  | 15 | 15 | 32.6% | 12 | 75.0% | 4 | 80.0% |
|  | 22 | 3 | 6.5% | 11 | 68.8% | 1 | 20.0% |
|  | 29 | 1 | 2.2% | 2 | 12.5% | - | - |
| 3 | 1 | 37 | 80.4% | 12 | 75.0% | 2 | 40.0% |
|  | 8 | 40 | 87.0% | 13 | 81.3% | 2 | 40.0% |
|  | 15 | 28 | 60.9% | 11 | 68.8% | 2 | 40.0% |
|  | 22 | 7 | 15.2% | 11 | 68.8% | 3 | 60.0% |
|  | 29 | 2 | 4.3% | 6 | 37.5% | 2 | 40.0% |

nal-IRI+5-FU/LV, liposomal irinotecan + 5-fluorouracil and leucovorin; GEM, Gemcitabine; GnP, Gemcitabine + nab-paclitaxel

eTable 4: Detail of QoL scores from EQ-5D-5L index values

1. NAL-IRI+5-FU/LV

| Cycle (C), Administration (A) and Day of answer (D) | NAL-IRI+5-FU/LV | | |
| --- | --- | --- | --- |
|  | N | Mean | SD |
| C2 A1 D1 | 46 | 0.803 | 0.142 |
| C2 A1 D2 | 44 | 0.804 | 0.192 |
| C2 A1 D4 | 42 | 0.748 | 0.194 |
| C2 A1 D6 | 42 | 0.678 | 0.247 |
| C2 A1 D8 | 44 | 0.691 | 0.189 |
| C2 A1 D11 | 42 | 0.757 | 0.150 |
| C3 A1 D1 | 37 | 0.776 | 0.197 |
| C3 A1 D2 | 37 | 0.783 | 0.149 |
| C3 A1 D4 | 37 | 0.765 | 0.152 |
| C3 A1 D6 | 37 | 0.729 | 0.181 |
| C3 A1 D8 | 40 | 0.723 | 0.182 |
| C3 A1 D11 | 37 | 0.757 | 0.205 |
| C4 A1 D1 | 28 | 0.782 | 0.159 |

nal-IRI+5-FU/LV, liposomal irinotecan + 5-fluorouracil and leucovorin

1. GnP or GEM

| Cycle (C), Administration (A) and Day of answer (D) | GnP | | | GEM | | |
| --- | --- | --- | --- | --- | --- | --- |
|  | N | Mean | SD | N | Mean | SD |
| C2 A1 D1 | 16 | 0.872 | 0.077 | 5 | 0.772 | 0.208 |
| C2 A1 D2 | 15 | 0.85 | 0.08 | 5 | 0.837 | 0.125 |
| C2 A1 D4 | 15 | 0.786 | 0.129 | 4 | 0.707 | 0.200 |
| C2 A1 D6 | 16 | 0.786 | 0.118 | 5 | 0.735 | 0.247 |
| C2 A2 D1 | 13 | 0.825 | 0.141 | 4 | 0.684 | 0.235 |
| C2 A2 D2 | 16 | 0.83 | 0.1 | 4 | 0.709 | 0.201 |
| C2 A2 D4 | 16 | 0.82 | 0.134 | 5 | 0.691 | 0.304 |
| C2 A2 D6 | 16 | 0.798 | 0.137 | 5 | 0.661 | 0.316 |
| C2 A3 D1 | 12 | 0.805 | 0.144 | 4 | 0.594 | 0.308 |
| C2 A3 D2 | 13 | 0.809 | 0.13 | 4 | 0.628 | 0.214 |
| C2 A3 D4 | 12 | 0.762 | 0.167 | 4 | 0.662 | 0.324 |
| C2 A3 D6 | 11 | 0.751 | 0.184 | 4 | 0.571 | 0.306 |
| C2 A3 D8 | 11 | 0.758 | 0.24 | 1 | 0.184 | - |
| C2 A3 D11 | 10 | 0.773 | 0.217 | 1 | 0.034 | - |
| C3 A1 D1 | 12 | 0.789 | 0.182 | 2 | 0.793 | 0.292 |
| C3 A1 D2 | 11 | 0.804 | 0.196 | 1 | 1 | - |
| C3 A1 D4 | 13 | 0.78 | 0.16 | 3 | 0.814 | 0.236 |
| C3 A1 D6 | 12 | 0.763 | 0.185 | 1 | 0.867 | - |
| C3 A2 D1 | 13 | 0.785 | 0.183 | 2 | 0.723 | 0.391 |
| C3 A2 D2 | 13 | 0.788 | 0.182 | 3 | 0.79 | 0.257 |
| C3 A2 D4 | 14 | 0.746 | 0.204 | 2 | 0.685 | 0.257 |
| C3 A2 D6 | 14 | 0.726 | 0.185 | 3 | 0.766 | 0.267 |
| C3 A3 D1 | 11 | 0.788 | 0.108 | 2 | 0.796 | 0.289 |
| C3 A3 D2 | 12 | 0.813 | 0.153 | 2 | 0.63 | 0.273 |
| C3 A3 D4 | 10 | 0.795 | 0.14 | 3 | 0.812 | 0.325 |
| C3 A3 D6 | 11 | 0.747 | 0.168 | 3 | 0.753 | 0.288 |
| C3 A3 D8 | 11 | 0.768 | 0.217 | 3 | 0.777 | 0.299 |
| C3 A3 D11 | 9 | 0.792 | 0.177 | 3 | 0.723 | 0.392 |
| C4 A3 D1 | 6 | 0.656 | 0.255 | 2 | 0.655 | 0.488 |

GnP, Gemcitabine + nab-paclitaxel; GEM, Gemcitabine

eTable 5: Detail of EORTC-QLQ C30 global health status

A) NAL-IRI+5-FU/LV

| Cycle (C), Administration (A) and Day of answer (D) | NAL-IRI+5-FU/LV | | |
| --- | --- | --- | --- |
|  | N | Mean | SD |
| C2 A1 D1 | 45 | 60.9 | 19.9 |
| C2 A1 D2 | 43 | 60.1 | 21.3 |
| C2 A1 D4 | 42 | 55.4 | 23.8 |
| C2 A1 D6 | 42 | 51.4 | 23.8 |
| C2 A1 D8 | 44 | 50.0 | 23.8 |
| C2 A1 D11 | 41 | 53.5 | 23.3 |
| C3 A1 D1 | 37 | 59.9 | 21.1 |
| C3 A1 D2 | 36 | 60.6 | 21.4 |
| C3 A1 D4 | 37 | 55.0 | 21.2 |
| C3 A1 D6 | 36 | 50.9 | 23.5 |
| C3 A1 D8 | 39 | 53.8 | 23.6 |
| C3 A1 D11 | 36 | 55.3 | 24.2 |
| C4 A1 D1 | 27 | 57.4 | 21.6 |

nal-IRI+5-FU/LV, liposomal irinotecan + 5-fluorouracil and leucovorin

B) GnP or GEM

| Cycle (C), Administration (A) and Day of answer (D) | GnP | | | GEM | | |
| --- | --- | --- | --- | --- | --- | --- |
|  | N | Mean | SD | N | Mean | SD |
| C2 A1 D1 | 16 | 70.8 | 18.5 | 5 | 62.5 | 22.0 |
| C2 A1 D2 | 15 | 67.8 | 16.6 | 5 | 55.0 | 20.1 |
| C2 A1 D4 | 15 | 65.6 | 19.1 | 4 | 41.7 | 21.5 |
| C2 A1 D6 | 16 | 64.1 | 18.4 | 5 | 50.0 | 26.4 |
| C2 A2 D1 | 13 | 63.5 | 20.6 | 4 | 43.8 | 15.8 |
| C2 A2 D2 | 16 | 63.5 | 19.5 | 4 | 35.4 | 4.2 |
| C2 A2 D4 | 16 | 67.7 | 20.4 | 5 | 46.7 | 25.4 |
| C2 A2 D6 | 16 | 64.1 | 27.5 | 5 | 35.0 | 30.8 |
| C2 A3 D1 | 12 | 64.6 | 21.1 | 4 | 33.3 | 6.8 |
| C2 A3 D2 | 13 | 62.8 | 23.5 | 4 | 37.5 | 10.8 |
| C2 A3 D4 | 12 | 59 | 22.3 | 4 | 47.9 | 23.9 |
| C2 A3 D6 | 11 | 55.3 | 20.8 | 4 | 29.2 | 10.8 |
| C2 A3 D8 | 11 | 52.3 | 24.7 | 1 | 16.7 | - |
| C2 A3 D11 | 10 | 54.2 | 25.5 | 1 | 0.0 | - |
| C3 A1 D1 | 12 | 59.7 | 27.3 | 2 | 45.8 | 5.9 |
| C3 A1 D2 | 11 | 63.6 | 23.1 | 1 | 41.7 | - |
| C3 A1 D4 | 13 | 58.3 | 24.3 | 3 | 55.6 | 24.1 |
| C3 A1 D6 | 12 | 64.6 | 17.5 | 1 | 41.7 | - |
| C3 A2 D1 | 13 | 64.7 | 17.1 | 2 | 33.3 | 11.8 |
| C3 A2 D2 | 13 | 59 | 24.2 | 3 | 52.8 | 26.8 |
| C3 A2 D4 | 14 | 58.9 | 25.4 | 2 | 37.5 | 5.9 |
| C3 A2 D6 | 14 | 61.3 | 26.3 | 3 | 50.0 | 28.9 |
| C3 A3 D1 | 11 | 61.4 | 19.1 | 2 | 37.5 | 5.9 |
| C3 A3 D2 | 12 | 60.4 | 18.5 | 2 | 33.3 | 11.8 |
| C3 A3 D4 | 10 | 61.7 | 18.9 | 3 | 52.8 | 26.8 |
| C3 A3 D6 | 11 | 54.5 | 22.2 | 3 | 50.0 | 30.0 |
| C3 A3 D8 | 11 | 53.8 | 23.7 | 3 | 52.8 | 26.8 |
| C3 A3 D11 | 9 | 54.6 | 26.7 | 3 | 47.2 | 33.7 |
| C4 A3 D1 | 6 | 45.8 | 34.1 | 2 | 29.2 | 17.7 |

GnP, Gemcitabine + nab-paclitaxel; GEM, Gemcitabine

eFigure 1: Proportion of participant with a reduction in QoL scores from EQ-5D-5L index values on day0 above MICD

1. Nal-IRI+5-FU/LV

MCID, Minimum clinical important difference

B) GnP

MCID, Minimum clinical important difference
